# Sensitive and Cost‐Effective Tools in the Detection of Ovarian Cancer Biomarkers

**DOI:** 10.1002/ansa.202400029

**Published:** 2024-10-24

**Authors:** Anis Elhami, Ahmad Mobed, Reza Soleimany, Yalda Yazdani, Esmat Sadat Kazemi, Mahya Mohammadi, Hossein Saffarfar

**Affiliations:** ^1^ Dentistry faculty Ahvaz Jundishapur University of Medical Sciences Ahvaz Iran; ^2^ Social Determinants of Health Research Center Tabriz University of Medical Sciences Tabriz Iran; ^3^ Faculty of Medicine Imam Reza Hospital Tabriz University of Medical Sciences Tabriz Iran; ^4^ Immunology Research Center Tabriz University of Medical Sciences Tabriz Iran; ^5^ Department of Obstetrics and Gynecology Alzahra Hospital Tabriz University of Medical Sciences Tabriz Iran; ^6^ Student Research Committee School of Medicine Shahid Beheshti University of Medical Sciences Tehran Iran; ^7^ Cardiovascular Research Center, Tehran Tehran University of Medical Sciences Tehran Iran

**Keywords:** apolipoprotein A1, CA125, human epididymis protein 4 (HE4), nanomedicine, ovarian cancer (OVC), transferrin, transthyretin

## Abstract

Women diagnosed with late‐stage ovarian cancer suffer a very high rate of mortality. Accordingly, it is imperative to detect and diagnose the disease as early as possible in its development. Achievement of this aim implies relatively large‐scale screening of women at an age of clinical significance through assay of biomarkers for disease present in blood or serum. Biosensor detection offers an attractive technology for the automated detection of such species. Among several biomarkers that have been identified that are present in patients with ovarian cancer, the only one that is commonly tested for in clinical use is cancer antigen 125, which is considered to be a poor biomarker for the disease. Here, we describe several biosensors that developed in the past decade for the detection of ovarian cancer biomarkers such as CA125, human epididymis protein 4 (HE4) and apolipoprotein A1. The challenges presented by the fabrication of biosensor devices for detecting ovarian cancer and the limited number of biosensors developed for this purpose are discussed.

**Abbreviations**
3‐APTES: 3‐Aminopropyl trimethoxy silane
AFP: alpha‐fetoprotein
CA125: cancer antigen 125
CV: cyclic voltammetry
AuNPs: gold nanoparticle
HGSC: high‐grade serous carcinoma
HE4: human epididymis protein 4
NIPAM: hydrophobic N‐isopropyl acrylamide
EIS: impedance spectroscopy
MI‐PPy NT: imprinted polypyrrole nanotubes
ITO‐PET: indium tin oxide‐polyethylene terephthalate
LOD: limit of detection
LPA: lysophosphatidic acid
SiNPs: mesoporous silica nanoparticles
MIP: molecularly imprinted polymer
OVC: ovarian cancer
VDP: polymerization
QDs: quantum dots
sEVs: small extracellular vesicles
SERS: surface‐enhanced Raman scattering
TVS: transvaginal sonography
SBMA: zwitterionic sulfobetaine

## 1 Introduction

Ovarian cancer (OVC) remains the deadliest of all gynaecological cancers, with most patients (58%) being diagnosed at a late stage (III or IV), leading to poor survival rates [1, 2]. Early detection is crucial, as survival rates exceed 90% in women diagnosed at stage I1, prompting international efforts to improve early detection over the past four decades [1, 2]. Despite this, about three‐quarters of invasive epithelial OVCs only present symptoms in the late stages, with early‐stage patients often experiencing only mild, non‐specific symptoms [3, 4]. This delayed onset of noticeable symptoms is significant because it often leads to late‐stage detection when the cancer is more advanced and difficult to treat. Consequently, the prognosis for patients diagnosed in the later stages is typically poorer, with reduced survival rates, emphasising the urgent need for improved early detection methods to enhance outcomes. An estimated 22% of patients with high‐grade serous carcinoma (HGSC) have germline mutations in the BRCA1/2 gene; consequently, understanding the role of these mutations is crucial for advancing early detection and personalised treatment strategies [3, 4]. Specifically, BRCA2 and BRCA1 mutations elevate the lifetime risk of OVC to 40%–60% and 11%–30%, respectively. Various factors, including childbearing, oral contraceptive use, tubal ligation surgery and hysterectomy, inversely correlate with OVC risk, while family history, hormone replacement therapy and obesity are recognised as additional risk factors [5, 6]. The challenge of delayed diagnosis due to the lack of specific symptoms and effective early detection biomarkers remains a significant issue in improving patient outcomes [5, 6]. Delayed diagnosis is a major factor in the poor prognosis of OVC and often occurs due to the lack of specific symptoms or effective biomarkers for early detection [7, 8]. Currently, the gold standard for OVC diagnosis involves histopathological examination, often necessitating surgical intervention, which carries inherent risks. Moreover, distinguishing between benign and malignant lesions preoperatively is crucial for appropriate management [7, 8]. Ovarian tumours are frequently first detected through transvaginal sonography (TVS), which, despite identifying features predictive of malignancy, has not yet achieved optimal diagnostic accuracy [9, 10]. Therefore, identifying reliable serum biomarkers is crucial for non‐invasive, cost‐effective and practical detection methods. The development of a two‐stage strategy using CA125 levels in conjunction with TVS has shown promise, as CA125 levels typically increase exponentially with cancer progression, unlike benign conditions [11, 12, 13]. The findings indicated several biomarkers, such as CA125, human epididymis protein 4 (HE4), apolipoprotein A1, transthyretin, transferrin and β2‐macroglobulin, could be considered as OVC indicators. Therefore, their fast and sensitive determination is valuable in OVC screenings and treatment plans. Here, we describe several biosensors that developed in the past decade for the detection of OVC biomarkers such as CA125, HE4 and apolipoprotein A1. The challenges presented by the fabrication of biosensor devices for detecting OVC and the limited number of biosensors developed for this purpose are discussed.

## 2 Ovarian Cancer and Related Biomarkers

OVC is not a single disease and can be divided into at least five different histological subtypes that have identifiable risk factors, cellular origin, molecular composition and characteristics [14, 15]. OVC is a global problem, often diagnosed at a late stage and without effective screening strategies. Standard treatments for newly diagnosed cancer include cytoreductive surgery and platinum‐based chemotherapy [14, 15]. Key biomarkers such as CA125, HE4 and BRCA1/2 mutations play a crucial role in the detection, prognosis and treatment of OVC [16, 17]. CA125 is a protein that is elevated in the blood of many women with OVC, making it a valuable marker for monitoring disease progression and treatment response, although it has limitations due to non‐specificity [16, 17]. HE4, another biomarker, is often used in conjunction with CA125 to improve diagnostic accuracy, particularly in differentiating between benign and malignant ovarian conditions. BRCA1/2 mutations are critical genetic markers that significantly increase the risk of OVC, especially HGSC [18, 19]. Identifying these mutations not only helps in assessing individual risk but also guides treatment strategies, as patients with BRCA mutations often respond better to PARP inhibitors, a targeted therapy [18, 19]. Together, these biomarkers enhance early detection, help predict prognosis and inform personalised treatment approaches, improving outcomes for OVC patients. Patients with BRCA mutations often respond well to PARP inhibitors, a targeted therapy that improves outcomes in those with hereditary OVC. Collectively, these biomarkers provide critical insights into early detection, risk assessment and personalised treatment strategies for OVC.

**FIGURE 1:**
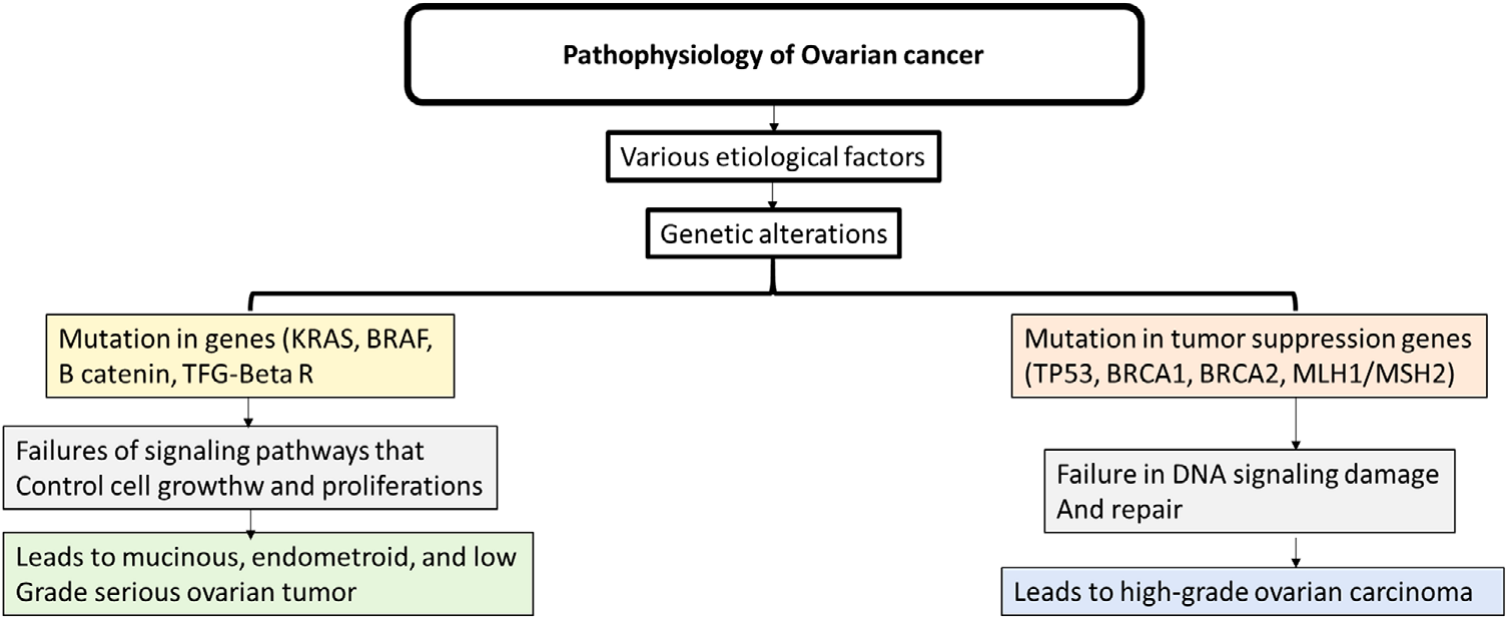
Pathophysiology of ovarian cancer. Ovarian cancer has been associated with penetrant germline pathogenic mutations in tumour suppressor genes, especially BRCA1 and BRCA2 adapted from ref. [28].

In cases of cancer relapse, antiangiogenic drugs, chemotherapy and poly(ADP‐ribose) polymerase inhibitors are used, and immunotherapies are presently being tested [20, 21]. HGSC is the most commonly diagnosed form of OVC and when diagnosed is often very responsive to platinum‐based chemotherapy [20, 21]. Today, no broadly acknowledged pathogenesis of OVC has been described. One of the leading problems in discovering the pathogenesis of OVC is the heterogeneous nature of OVC, which includes many histological types with various behaviour and characteristics [22, 23]. Even though 40% of ovarian tumours are non‐epithelial, only 10% of OVCs are non‐epithelial. Initially, it was thought that all OVCs arose from the surface epithelium of ovarian cells. During ovulation, these surface epithelial cells experience physical injury and are immediately restored [22, 23]. During a woman's life cycle, ovulation occurs continuously, causing repeated damage to the epithelium, eventually causing damage to the cell's DNA. Epithelial cells with damaged DNA are susceptible to changes, facilitating invasion of the cortical stroma [24, 25]. This invasion eventually becomes trapped and forms a sphere of epithelial cells in the lamina propria called a cortical cyst. In the ovary, epithelial cells are exposed to ovarian hormones that stimulate cell proliferation, turning into cancer cells [24, 25]. The pathophysiology of OVC involves complex interactions between genetic, environmental and hormonal factors, leading to the uncontrolled growth of cells in the ovaries. OVC typically originates in the epithelial cells that line the surface of the ovary, although it can also arise from germ cells or stromal cells. For example, mutations in tumour suppressor genes such as BRCA1 and BRCA2, as well as other genes like TP53 and PTEN, play a critical role (Figure 1) [26, 27]. These mutations disrupt the normal regulation of cell division, DNA repair and apoptosis, allowing abnormal cells to proliferate. Figure 1 illustrates several key genetic factors.

Molecular pathways in this process include intracellular effectors involved in malignant transformation, such as VEGF, nitric oxide synthase, NF‐κB and cyclooxygenase‐2. Other inflammatory chemicals, such as asbestos and talc, have also been shown to cause OVC [28, 29, 30]. However, the association between exposure to talc or asbestos and the development of OVC has not been confirmed in animal models [28, 29, 30].

## 3 Ovarian Cancer Detection Approaches

Regular screening for early detection has been shown to be very beneficial for many forms of cancer [31, 32]. For example, since the introduction of the Papanicolaou (Pap) test, cervical cancer incidence and mortality in screened individuals in the United States have decreased by more than 75% [31, 32]. Correspondingly, colonoscopy screening has been shown to be associated with a 70% reduction in the risk of death from colorectal cancer [33, 34]. Currently, available clinical methods for colorectal cancer screening are primarily limited to physical assessment by the clinician, adnexal imaging with transvaginal ultrasound (TVU) and measurement of serum levels of cancer antigen biomarker protein 125 (CA125) [35, 36]. Ultrasound transmission velocity is the most commonly used imaging modality to detect OVC and allows clinicians to identify abnormalities in the size and shape of ovarian tissues [35, 36]. Images obtained by TVU are reviewed by radiologists to evaluate the presence of specific clinical features based on the simple rules of the International Ovarian Tumor Analysis (IOTA) [37, 38]. Some of the features evaluated include the presence of papillae, ascites and/or internal blood flow, which are ultimately used to predict the likelihood and stage of malignancy [37, 38]. Despite this reality, the clinical need for accurate OVC screening and diagnosis remains urgent. Likewise, many clinicians and researchers have attempted to develop new methods to identify early‐stage diseases from a variety of biomarker sources. Therefore, as demonstrated, serum biomarkers may play an important role in the detection and management of OVC. Therefore, the identification of OEV‐associated biomarkers such as CA125, HE4, apolipoprotein A1, transthyretin, transferrin and β2‐macroglobulin may be promising for the detection, screening and OVC treatment. Over the past decade, different methods have been developed to detect biomarkers in serum. Among other things, due to their high sensitivity, affordable price and other advantages, biosensors have attracted the interest of researchers. The next part of the article is dedicated to the overview of biosensor technology and a summary of recent OVC biosensors.

## 4 Biosensor Methods

Construction of biosensors, transduction devices, materials and immobilisation approaches involves multidisciplinary investigation in biology, chemistry and engineering [39, 40]. Materials used in biosensors are classified into three groups based on their mechanism: the microbial group contains microorganisms, the biocatalytic group includes enzymes and the bioaffinity group includes antibodies and nucleic acids [39, 41]. The biosensor designed for this work must not rely on specific physical parameters such as temperature or pH, as maintaining sensitivity is a priority. This ensures that external conditions do not interfere with the sensor's performance, unlike other biosensors, which may be intentionally designed to measure these factors [42, 43].

The term ‘biosensor’ was coined by Cammann, and its definition was formalised by IUPAC. A biosensor generally comprises three key components: a bioreceptor (such as an enzyme, antibody, cell, nucleic acid or aptamer), a transducer (often made from semiconducting or nanomaterials) and an electronic system that includes a signal amplifier, processor and display unit [44].

**FIGURE 2:**
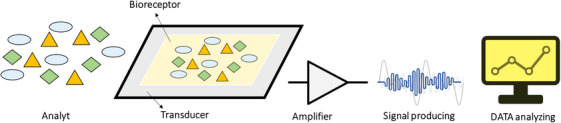
Schematic illustration of biosensor device. Typically, biosensors are comprised of three components: (1) the detector, which identifies the stimulus; (2) the transducer, which converts this stimulus to a useful output; and (3) the signal processing system, which involves amplification and display of the output in an appropriate format.

**FIGURE 3:**
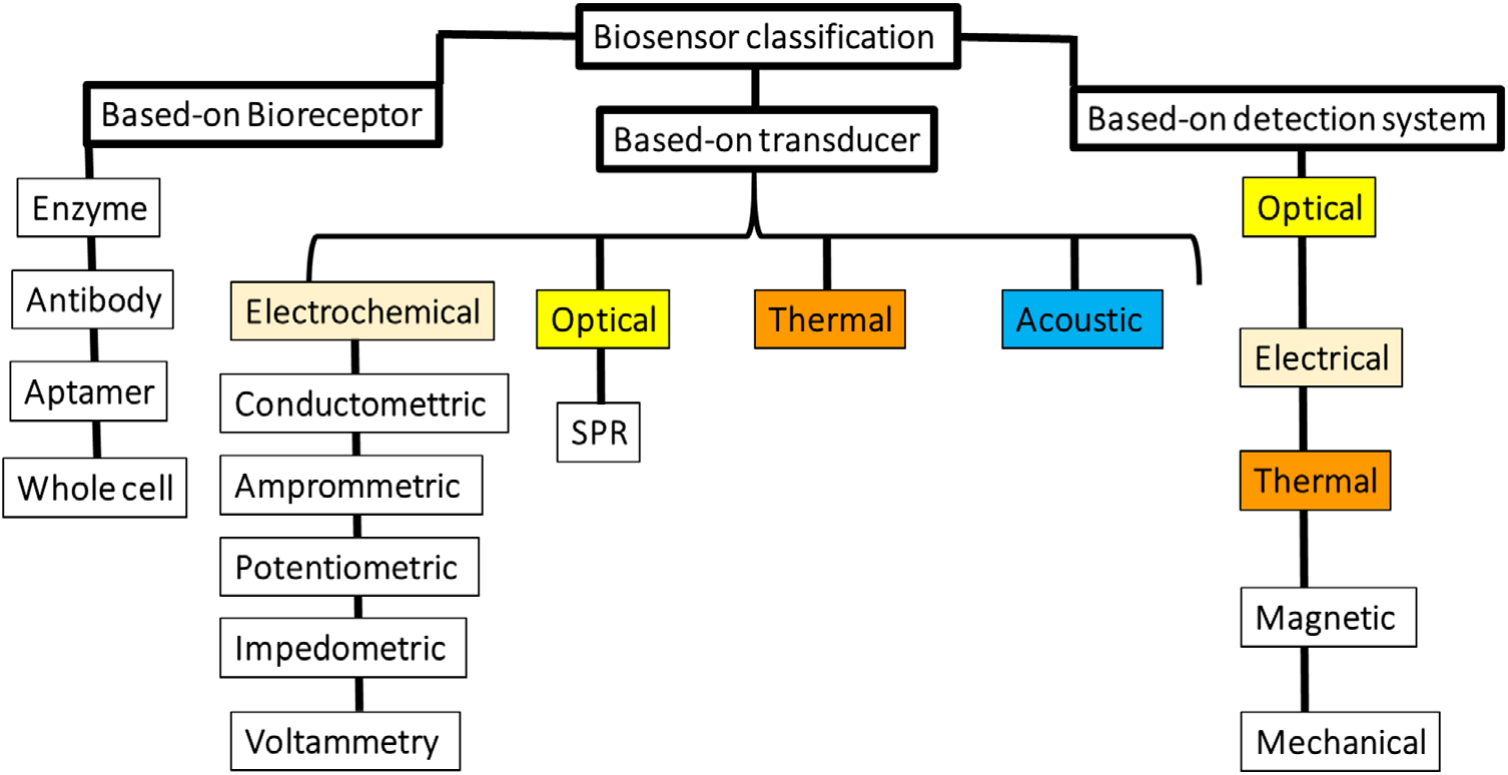
Biosensors classification. Biosensors may be classified according to the biological specificity conferring mechanism, the mode of signal transduction or, alternatively, a combination of the two. These might also be described as amperometric, potentiometric and field‐effect or conductivity sensors.

A successful biosensor mainly consists of two main components: a biological receptor or sensing element and a transducer [45, 46]. Typically, a signal processing unit, including a display or printer, is used in conjunction with the biosensor, as shown in Figure 2. As discussed in the above‐mentioned sections, a biosensor consists of a biological receptor coupled to a transducer and a signal processor, thus operating based on signal transduction. The arrangement of these ingredients is planned to convert a biological response into a corresponding electrical response and eventually a quantifiable result [45, 46]. In simpler terms, a biosensor is responsible for the measurable analysis of a molecule by linking its biological activity to a quantifiable signal. Biosensors are classified into three main groups based on bioreceptors, transducers and detection systems (Figure 3).

The method of transmission depends on the physicochemical changes caused by the sensor element. Then, based on various types of probes, biosensors can be electrochemical (ammeter, conductivity and potentiometry), optical (absorbance, chemiluminescence and fluorescence), piezoelectric (acoustic and ultrasonic) and calorimetric [47, 48]. Biosensors can also be arranged to reveal, ordered by initial, which is the simplest approach, which involves direct detection of the increase of an enzymatically produced element or the reduction of a substrate of a redox chemical using a specific intermediate for electron exchange. Glucose biosensors using the chemical glucose oxidase and oxygen recognise decreased oxygen levels or increased hydrogen peroxide levels relative to glucose levels [47, 48]. In biosensor technology, nanoparticles play key roles. The performance differences between sensors that use NPs and those that do not can be quite significant. For example, sensors that incorporate nanoparticles often exhibit enhanced sensitivity and lower detection limits [49, 50]. NPs can amplify the signal due to their large surface area‐to‐volume ratio and the ability to bind more target molecules, which improves the sensor's overall sensitivity [49, 50]. Additionally, NPs can contribute to faster response times due to their high surface area, which allows for quicker interactions with the analyte. For example, in electrochemical sensors, NPs can enhance electron transfer rates. Moreover, functionalisation of NPs with specific ligands or antibodies can improve specificity by selectively binding to the target analyte while reducing interference from other substances [51, 52]. In summary, nanoparticles can significantly improve the performance of sensors by enhancing sensitivity, reducing detection limits, and sometimes accelerating response times. The choice of nanoparticles is typically guided by the specific requirements of the application, such as the need for high sensitivity, selectivity or particular interaction properties [52, 53].

Before the recent advances, OVC biosensors primarily relied on the detection of traditional biomarkers such as CA125 and HE4. These biomarkers were typically measured using enzyme‐linked immunosorbent assays (ELISA) and other similar methods, which provided some level of specificity but lacked the sensitivity needed for early‐stage detection. CA125, in particular, has been widely used in clinical practice for monitoring disease progression and recurrence, but it has limitations due to its elevated levels in non‐cancerous conditions and its lower sensitivity in detecting early‐stage OVC. Similarly, HE4 was introduced as an adjunct to CA125 to improve diagnostic accuracy, but it still failed to address the challenge of early detection effectively. These conventional biosensors, while valuable, were often influenced by external factors such as pH and temperature and required large sample volumes, expensive reagents and laboratory infrastructure. This background paved the way for the development of newer biosensors that leverage nanotechnology, microfluidics and electrochemical detection methods, which offer improved sensitivity, specificity and portability for point‐of‐care applications. Recent advances have focused on addressing the limitations of traditional biosensors, providing a foundation for more accurate and early detection of OVC.

## 5 Recent Advances in Ovarian Cancer Biosensors

In biosensor technology, it is important to elaborate on the desirable limits of detection (LODs) for each biomarker discussed, as this provides a foundation for understanding the effectiveness of current biosensors. For instance:

- CA125: A desirable LOD for CA125 should be in the low nanogram per millilitre (ng mL^−1^) range, as elevated CA125 levels in OVC patients are typically above 35 U mL^−1^. Sensors with a lower LOD can detect early‐stage cancers when CA125 levels are still relatively low, improving early diagnosis [54].
- HE4: The LOD for HE4 should also fall in the low nanogram per millilitre (ng mL^−1^) range, with a threshold of 150 pmol L^−1^ commonly used in clinical settings. A sensor capable of detecting HE4 at or below this level could effectively aid in diagnosing OVC, particularly when combined with CA125 [55].
- BRCA1/2 mutations: For genetic biomarkers like BRCA1/2, sensitivity to even small amounts of mutated DNA or RNA is crucial. LOD for these sensors should reach the picomolar (pM) or even femtomolar (fM) range to detect minute quantities of circulating tumour DNA (ctDNA) or gene mutations associated with OVC, enabling early detection and personalised treatment planning [56].

A highly specific and sensitive aptamer‐based SPR optical platform was advanced for the detection of CA125 antigen. This work used a streptavidin‐coated gold chip by affinity capture to immobilise biotinylated CA125 aptamer (Ap) [57]. An innovative label‐free electrochemical biosensor by magnetically induced self‐assembly (Mg_0.5_Cu_0.5_Fe_2_O_4_‐Au) was proposed for the detection of CA125 [58]. CuCo‐ONSs@AuNPs nanocomposites were assembled in an ultra‐sensitive immunosensor for the detection of CA125. The developed platform showed acceptable LOD and linearity [59]. Simultaneous detection is performed by three different electroactive nanomaterials, namely CdTe, gold nanoparticles (AuNPs) and PbS core‐like quantum dots (QDs), conjugated to three specific antibodies. Mesoporous silica nanoparticles (SiNPs) were integrated into a nanocomposite material for higher electroactive label loading, leading to significant enhancement of the electrochemical signal [60]. Label‐free disposable electrochemical immunobiosensor designed for rapid determination of HE4 and CA125. The nanosensors are designed to be easy to use and can be used in point‐of‐care testing to quickly and conveniently detect CA125 and HE4 with high selectivity, sensitivity and repeatability [61]. A label‐free electrochemical dual CA125‐HE4 immunosensor was fabricated for the sensitive, rapid and practical immediate determination of CA125 and HE4. This biosensor was disposable, dual screen‐printed carbon electrodes modified with rGO, polythionine and AuNPs. The developed dual immunosensors showed acceptable potential in the POC test's simultaneous determination of CA125 and HE4 with high sensitivity, selectivity and repeatability [62]. The MI‐PPy NT@Au‐SPE electrochemical chemosensor (molecular sensor) based on the molecularly imprinted polymer (MIP) method was advanced for the highly selective and sensitive determination of CA125. The settled chemosensors, with their original design combined with a simplistic fabrication method, demonstrate to be promising as future state‐of‐the‐art biosensors [63]. An antifouling electrochemical biosensor was established for the detection of CA125 in clinical serum samples based on copolymerisation zwitterionic sulfobetaine (SBMA) and hydrophobic N‐isopropyl acrylamide (NIPAM). The advanced platform exhibited biological stability and good biocompatibility and the PSN modified sensing surface realised a long‐term antifouling for 15 days in a buffer solution [64]. An affinity‐based electrochemical biosensor through antifouling properties for the detection of lysophosphatidic acid (LPA) was engineered [65]. Carbon ink/carbon dot/zine oxide (C‐ink/CD/ZnO) nanocomposite was employed for the sensitive detection of CA125. The created immunosensor showed strong biocompatibility, chemical stability, high conductive signal and accuracy [66]. NiFe_2_O_4_ magnetic nanoparticles were settled for the diagnosis of CA125. The immunosensor showed excellent stability, reproducibility, specificity and selectivity. These results are of great importance for the development of new high‐performance point‐of‐care electrochemical biosensors for application in the detection of CA125 antigen, a widely used biomarker to predict OVC [67]. Small extracellular vesicles (sEVs) are double‐layered lipid vesicles that carry important molecules (e.g., proteins, DNA, RNA and lipids) for intercellular communication, considered as promising biomarkers to diagnose cancer. On the other hand, the detection of sEVs remains challenging due to their unique characteristics, such as size and phenotypic heterogeneity. Recently, an innovative surface‐enhanced Raman scattering (SERS) assay biosensor was improved for sEV detection [68]. Magnetic nanocomposites (g‐C_3_N_4_/MoS_2_/Fe_3_O_4_) on a glassy carbon electrode (GCE) was developed as a label‐free aptasensor for detection of CA125 [69]. An innovative nanocomposite (g‐C_3_N_4_/Fe_3_O_4_/PANI) was developed as an electrochemical aptasensor for detecting 125 biomarkers. This work uses SWV and electrochemical impedance spectroscopy techniques for rapid and sensitive detection [70]. Some studies indicate that CA125 and the biomarker mesothelin may manifest with elevated serum OVC. However, these biomarkers also show elevated serum levels in diseases such as colon cancer and mesothelioma. A surface plasmon resonance (SPR) biosensor was improved for simultaneous detection of CA125 and mesothelin using AuNPs [71]. EpCAM and CD24 are two important overexpressed proteins in OVC. A dual‐aptamer targeted exosome‐based approach was planned to detect EpCAM and CD24 [72]. Cu(II)‐Polyethyleneimine (PEI)/Silica Nanoparticle@Overoxidized Polypyrrole (OPPy)/Multi‐Walled Carbon Nanotubes (MWNTs) Hybrid Nanomaterial was employed for ultra‐sensitive detection of CA125 [73]. A novel optical probe Tb‐acetylacetone (Tb‐ACAC) doped with improved epoxy cellulose polymer immobilised with the monoclonal antibody CA125 provides a precise and highly selective method for early diagnosis of OVC by detecting CA125 in serum samples. The approach utilises the quenching of Tb‐ACAC emission upon binding to CA125 [74]. HE4) is a protein that is overexpressed in OVC but not in healthy or benign diseases. HE4 in urine is a biomarker with high stability and diagnostic value in the detection of OVC. Recently, aptamers have emerged as low‐cost detection probes for cancer detection. Aptamers are single‐stranded oligonucleotides that bind to target molecules with high affinity. Recently, a DNA aptamer with nanomolar range affinity targeting human HE4 in urine was selected, identified and characterised [75]. A signal‐on electrochemical aptasensor was proposed for sensitive detection of HE4 based on functionalised metal‐organic framework/Ketjenblack nanocomposite. In this research, a metal‐organic framework/Ketjenblack (NH_2_‐MIL‐53(Al)/KB) composite with a large specific surface area and copious amino groups was organised as a signal amplifier to anchor AuNPs [76]. A disposable immunosensor was constructed for highly sensitive detection of HE4 in human serum samples. In this work, an impedimetric biosensor was invented for the analysis of HE4 by modifying the surface of a one‐use ITO‐PET page with a certain dimension by 3‐APTES [77]. An original electrochemical immunosensor was proposed for sensitive and selective detection of the apolipoprotein A4 as an important biomarker in different diseases [78]. An electrochemical aptasensor was fabricated for sensitive and specific detection of A4. The developed platform allows concurrent quantification of apoE‐HDL‐C, apoE‐HDL and HDL‐C on the same stage, proposing a convenient, efficient and purification‐free sensing approach for prognostic disease biomarkers [79]. Human A4 was detected by a label‐free electrochemical biosensor based on proximity hybridisation activated rolling circle amplification. Furthermore, this method has the added advantage of simplicity and low cost, as it avoids both the costly labelling process and the complicated probe fixation process. The positive results indicate that the proposed sensor has promising potential in the clinical diagnosis of depression [80].

Table 1 provides a detailed comparison of various biosensors developed for detecting OVC biomarkers, highlighting their methodologies, types and performance metrics. The biosensors listed include aptamer and immunosensors utilising a range of techniques such as electrochemical methods, surface‐enhanced Raman spectroscopy (SERS) and optical approaches. Each biosensor is evaluated based on its linearity range and LOD, reflecting its sensitivity and suitability for clinical use. For instance, immunosensors using advanced nanoparticles (NPs) like g‐C_3_N_4_/Fe_3_O_4_/PANI and AuNPs show high sensitivity with LODs as low as 0.298 U mL^−1^ and 0.1 fg mL^−1^, respectively, while aptasensors exhibit LODs in the femtomolar to picomolar range for biomarkers like CA125 and HE4. This diversity in detection limits and methodologies underscores the ongoing advancements in biosensor technology aimed at improving early detection and monitoring of OVC. Additionally, the table reveals the evolving complexity and performance improvements in biosensor design, from basic electrochemical sensors to sophisticated optical and SERS‐based approaches, reflecting significant progress in the field.

**TABLE 1:** Summary of recent advanced OVC biosensors analytical properties and techniques.

Biomarkers	Type	Methods	NPs	Sample/Matrix	Linearity	LOD	Refs.
CA125	Aptamer	Electrochemical	—	Serum	10–100 U mL^−1^	0.01 U mL^−1^	[57]
CA125	Immunosensor	Electrochemical DPVs	Mg_0.5_Cu_0.5_Fe_2_O_4_‐Au	Serum	5–125 U mL^−1^	4.4 U mL^−1^	[58]
CA125	Immunosensor	Electrochemical DPVs	CuCo‐ONSs@AuNPs	Serum	1 × 10^−7^ to 1 × 10^−3^ U mL^−1^	3.9 × 10^−8^ U mL^−1^	[59]
HE4, AFP CA125	Immunosensor	Electrochemical DPVs	QDs and SiNPs‐AuNPs	Serum	0.02–20 pM for HE4, 0.45–450 IU L^− 1^ for CA125 and 0.1–500 ng L^−1^	20 pM for HE4, 450 IU L^−1^ for CA125, and 500 ng L^−1^	[60]
HE4, CA125	Immunosensor	Electrochemical DPVs	rGO, AuNPs	Blood serum	0–50 ng mL^− 1^, and 50–500 ng mL^− 1^	100 pg mL^− 1^	[61]
HE4, CA125	Immunosensor	Electrochemical CVs, DPV and SWV	rGO, AuNPs	Blood serum	1–100 pg mL^−1^ and 1–50 ng mL^−1^	50 ng mL^−1^	[62]
CA125	Chemosensor	MIP, CVs	MI‐PPy NT@Au‐SPE	Serum	0.1–100 U mL^−1^	0.4 U mL^−1^	[63]
CA125	Immunosensor	DPVs	PANI/GCE‐ SBMA‐ NIPAM	Blood serum	0.01–1000 U mL^−1^	2.7 mU mL^−1^	[64]
LPA	Immunosensor	Electrochemical	—	Goat serum	0.01–10 µM	0.7 µM	[65]
CA125	Immunosensor	Electrochemical	C‐ink/CD/ZnO	Clinical	1 ag mL^−1^ to 100 ng mL^−1^	0.1 fg mL^−1^	[66]
CA125	Immunosensor	Electrochemical	NiFe_2_O_4_	Clinical	—	—	[67]
sEVs	Optical	SERS	—	Plasma	—	1.5 × 10^5^ particles per µL	[68]
CA125	Aptasensor	Electrochemical	g‐C_3_N_4_/MoS_2_/Fe_3_O_4_	Serum	2–10 U mL^−1^	0.202 U mL^−1^	[69]
CA125	Immunosensor	Electrochemical, SWV	g‐C_3_N_4_/Fe_3_O_4_/PANI	Serum	—	0.298 U mL^−1^	[70]
CA125 and mesothelin	Immunosensor	SPR	AuNPs	Serum	—	3.03 and 13.62 nM	[71]
EpCAM and CD24	Aptasensor	Optical	—	Serum	—	—	[72]
CA125	Immunosensor	Electrochemical, SWV	Cu^2+^/PEI/SiO_2_ NPs@ OPPy/MWNTs	Serum	0.001–500 U mL^−1^	3.4 × 10^−4^ U mL^−1^	[73]
CA125	Immunosensor	Electrochemical, SWV	Tb–acetylacetone (Tb–ACAC)	Serum	—	—	[74]
HE4	Aptasensor	Optical	HisPur Ni‐NTA beads	Serum	127 ± 28 nM	—	[75]
HE4	Aptasensor	Optical	NH_2_‐MIL‐53(Al)/KB‐AuNPs	Serum	1–10 nM	0.41 fM	[76]
HE4	Immunosensor	Electrochemical, CVs, EIS	ITO‐PET/3‐APTES	Serum	1 and 3000 pg mL^−1^	0.3134 pg mL^−1^	[77]
Apo‐A4	Immunosensor	Electrochemical, SWV	ZIF‐8@N‐Gr	Serum	1.47 × 10^−10^ to 3.00 × 10^−7^ g mL^−1^	8.33 × 10^−11^ g mL^−1^	[78]
Apo‐A4	Immunosensor	Electrochemical	—	Serum	0–250 mg dL^−1^	100 mg dL^−1^	[79]
Apo‐A4	Aptasensor	Electrochemical		Serum	1 pg mL^−1^ to 100 ng mL^−1^	0.51 pg mL^−1^	[80]

## 6 Conclusion and Future Prospective

Rapid and sensitive recognition of OVC is the basic principle leading to success in cancer management. To date, many biosensors have been reported in the literature only for the determination of CA125. There are few reports on the recognition of other important biomarkers such as HE4 and Apo‐A4. Therefore, considerable research is required to fabricate a biosensor for the determination of HE4 and prostaglandin. Furthermore, ELISA is currently the only commercially available identification kit for detecting OVC biomarkers with a narrow detection range and low sensitivity. To eliminate these problems, nanomaterial‐based biosensors have an advantage over ELISA because they can attach to aptasensors, displaying enhanced sensitivity and selectivity. On the other hand, although optical biosensors have yielded favourable analytical results, most of them have complex and tedious arrangements, often failing to provide a simple method for identifying biomarkers. To solve this problem, magnetoresistive, electrical or electrochemical sensors have great potential and offer many advantages. Also, few reports have been presented on wearable sensors used outside the laboratory to identify OVC biomarkers. On the other hand, to commercialise biosensor platforms at the device level is essential and is just the beginning. To address this issue, existing research and progress in developing cost‐effective paper‐based disposable biosensors or FET, and lab‐on‐chip microfluidic devices for identifying OVC biomarkers shows great promise. In this regard, the most recent related articles were studied, and the corresponding analytical performance of the detection mechanisms for different biosensors was evaluated. In summary, exciting advances are expected to take place soon in terms of detection methods and reduced testing kits, which will open up a new avenue for OVC patients. In conclusion, the early detection of OVC remains a significant challenge due to the late presentation of symptoms and the current limitations in effective biomarkers. Despite advances in diagnostic methods, such as TVS and serum biomarker testing, there is a crucial need for improved, non‐invasive screening tools that offer high sensitivity and specificity. Recent developments in biosensor technology hold promise for overcoming these challenges by providing rapid and accurate detection of key biomarkers like CA125, HE4 and others. Future research should focus on enhancing the performance of these biosensors, exploring novel biomarkers, and integrating these technologies into comprehensive screening strategies. By advancing biosensor technology and addressing current limitations, we can improve early detection rates and ultimately enhance survival outcomes for OVC patients.

## Conflicts of Interest

The authors declare no conflicts of interest.
